# Neuroprotective Riluzole-Releasing Electrospun Implants for Spinal Cord Injury

**DOI:** 10.1021/acs.molpharmaceut.4c01270

**Published:** 2025-05-16

**Authors:** Mathilde M. Ullrich, Bhavana Pulipaka, Jing Yin, Jana Hlinková, Fangyuan Zhang, Michael W. Chan, Fergal J. O’Brien, Adrian Dervan, Karolina Dziemidowicz

**Affiliations:** † Department of Pharmaceutics, UCL School of Pharmacy, 29-39 Brunswick Square, London WC1N 1AX, U.K.; ‡ Tissue Engineering Research Group, Department of Anatomy & Regenerative Medicine, Royal College of Surgeons in Ireland (RCSI), 123 St Stephens Green, Dublin 2 D02 YN77, Ireland; § Department of Tissue Engineering, 48311 Institute of Experimental Medicine of the Czech Academy of Sciences, Videnska 1083, Prague 142 20, Czechia; ∥ Department of Biomaterials, Faculty of Dentistry, University of Oslo, Geitmyrsveien 69/71, PB 1142 Blindern, Oslo 0317, Norway; ⊥ Advanced Materials and Bioengineering Research Centre (AMBER), Trinity College Dublin, College Green, Dublin 2 D02 W9K7, Ireland

**Keywords:** electrospinning, spinal cord injury, riluzole, SH-SY5Y, glutamate-induced excitotoxicity

## Abstract

Spinal cord injury (SCI) results in paralysis, driven partly by widespread glutamate-induced secondary excitotoxic neuronal cell death in and around the injury site. While there is no curative treatment, the standard of care often requires interventive decompression surgery and repair of the damaged dura mater close to the injury locus using dural substitutes. Such intervention provides an opportunity for early and local delivery of therapeutics directly to the injured cord via a drug-loaded synthetic dural substitute for localized pharmacological therapy. Riluzole, a glutamate-release inhibitor, has shown neuroprotective potential in patients with traumatic SCI, and therefore, this study aimed to develop an electrospun riluzole-loaded synthetic dural substitute patch suitable for the treatment of glutamate-induced injury in neurons. A glutamate-induced excitotoxicity was optimized in SH-SY5Y cells by exploring the effect of glutamate concentration and exposure duration. The most effective timing for administering riluzole was found to be at the onset of glutamate release as this helped to limit extended periods of glutamate-induced excitotoxic cell death. Riluzole-loaded patches were prepared by using blend electrospinning. Physicochemical characterization of the patches showed the successful encapsulation of riluzole within polycaprolactone fibers. A drug release study showed an initial burst release of riluzole within the first 24 h, followed by a sustained release of the drug over 52 days to up to approximately 400 μg released for the highest loading of riluzole within fiber patches. Finally, riluzole eluted from electrospun fibers remained pharmacologically active and was capable of counteracting glutamate-induced excitotoxicity in SH-SY5Y cells, suggesting the clinical potential of riluzole-loaded dural substitutes in counteracting the effects of secondary injury in the injured spinal cord.

## Graphical abstract

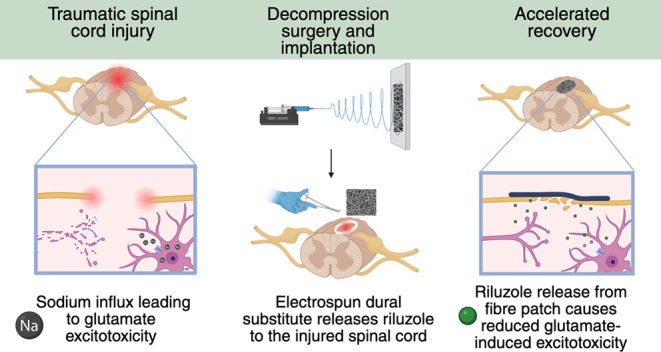

## 1 Introduction

Traumatic spinal cord injury (SCI) is a devastating neurological disorder characterized by significant tissue damage and a cascade of pathophysiological events that contribute to loss of function and long-term disability.^1^ Globally, the rates for traumatic SCI range between 20 and 45 cases per million people.^2^ At present, the standard of treatment is emergency decompression surgery performed immediately after the trauma.^3^ During this procedure, the dura mater, which is the tough outer connective tissue layer of the meningeal sheath, is often torn. If left untreated, this can lead to persistent cerebrospinal fluid (CSF) leakage and associated intracranial hypotension, increased risk of infection, delayed wound healing, and neurological dysfunction.^4^

Dural substitutes can be used to seal the subdural environment and protect the injured spinal cord from further damage and prevent infection.^5^ An ideal dural substitute must be mechanically strong to withstand handling during surgery but flexible enough to allow easy manipulation and tight suturing to the damaged dura mater. Furthermore, it should mimic the natural dura, facilitate cell infiltration, and be gradually replaced by connective tissue.^5^ While the gold standard is still the use of autologous connective tissues harvested from elsewhere in the body (e.g., fascia lata), several studies have explored the use of alternative dural substitutes, for example, from porcine connective tissues such as heart pericardium, intestinal peritoneum, and submucosa.^6^ Although showing some promise, the potential issue of disease transmission limits the rapid translation of such xenotissues to the clinic.^6,7^ In contrast, synthetic polymers avoid such limitations and can be tailored to suit the physico-mechanical properties of the target tissue.^8^ Recent studies have explored the use of polycaprolactone (PCL)-based patches, which have demonstrated the ability to carry and release therapeutic agents.^9–11^ Such wound-sealing patches can be engineered to be stitched into the dura, while simultaneously delivering therapeutics to the injured spinal cord, hence offering a dual function.^10^

Pharmacological treatment of SCI is currently restricted to a course of methylprednisolone, a glucocorticoid immunosuppressant, facilitating reduction in inflammation in the injury site,^11^ although its use has been questioned.^12^ At present, no therapy actively seeks to alleviate the progressive neurodegeneration in and around the injury site as damaged neurons slowly succumb to secondary injury-mediated pathophysiological events.^13^ Emerging treatments, such as riluzole, a neuroprotective agent currently licensed for amyotrophic lateral sclerosis, have shown promise for the treatment of SCI by targeting pathological sodium influx and abnormal glutamatergic neurotransmission^14^ contributing to neuronal hyperexcitability and subsequent excitotoxic death.^15^ Preclinical studies indicate that riluzole can improve neurological outcomes in SCI models,^16^ leading to further evaluation in ongoing phase II/III clinical trials (NCT00876889).^14^ However, systemic administration has been associated with limited efficacy and adverse effects such as gastrointestinal disturbances, fatigue, and malaise,^17^ which could be potentially overcome by delivering the drug directly to the site of action.

A localized delivery of riluzole to the injured spinal cord could be achieved using drug-loaded implants prepared with electrospinning.^18^ In this method, solid nano- or micro-sized fibers are produced from natural and synthetic polymers^19,20^ through the application of an electric field. Therapeutic agents can be blended into the polymer solution to produce drug-loaded fibers, which are then collected to form a patch that can serve as a scaffold for tissue engineering and drug delivery applications.^21^ Electrospun dural substitutes have been explored due to their tunable mechanical properties, high surface-to-volume ratio, and ease of surface modification.^22^ Their fibrous solid structure is similar to the extracellular matrix, thereby facilitating tissue regeneration^23^ and providing a robust scaffold for nerve regrowth—a structural support that soft materials, such as hydrogels, inherently lack.^18^

In this study, we describe the successful production of riluzole-loaded electrospun fiber patches. PCL was used to encapsulate riluzole due to its biocompatibility, biodegradability,^24^ and favorable mechanical properties.^25^ Their pharmacological function was assessed in an optimized in vitro SH-SY5Y cell-based glutamate-induced excitotoxicity model, where patches loaded with as little as 0.25–1% w/v riluzole were found to promote neural recovery. To the best of our knowledge, this is the first study reporting electrospun implants capable of rescuing SH-SY5Y cells from glutamate-induced damage.

## 2 Materials and Methods

### 2.1 Electrospinning Parameters

Electrospun fibers were manufactured using a NEU-BM (Tong Li Tech, China) electrospinning instrument. PCL (Mw ∼ 80 kDa, Sigma-Aldrich, UK) was dissolved in a 90% v/v solution of hexafluoro-2-propanol (HFIP, Sigma-Aldrich, UK) in deionized water (dH_2_O) and stirred overnight. Riluzole (98%, Thermo Fisher, UK) at various drug loadings (see Table 1 for full details) was added to the polymer solution and vortexed until all drug was dissolved. 2.5 mL of the polymer–drug solution was then loaded into a 5 mL syringe attached to a size 9 (0.6 mm inner diameter) metal spinneret needle (Tong Li Tech, China) and ejected at a flow rate of 2 mL/h. The fibers were collected on baking paper on a 9 cm diameter mandrel at a 14 cm distance from the spinneret. The spinneret was scanning parallel to the collector over a distance of 25 mm at a speed of 50 mm/s and spinning at 500 rpm. The negative voltage applied to the collector was constant at −3 kV, while the positive charge at the spinneret ranged between 10 and 12 kV. All electrospinning experiments were conducted at room temperature (18 °C–23 °C) and at 25 ± 5% relative humidity.

**Table 1:** Summary of Final Optimized Electrospinning Parameters Used to Produce Riluzole-Loaded Fibers

solution composition	10% w/v PCL in HFIP/dH_2_O (9:1) + 0%, 0.25%, 0.5%, or 1% w/v riluzole
solution volume	2.5 mL
flow rate	2 mL/h
voltage	10 kV–12 kV, mandrel voltage: –3 kV
distance between the needle and collector	14 cm
rotating mandrel speed	500 rpm
needle inner diameter	0.6 mm
temperature	18 °C–23 °C
humidity	25 ± 5%

### 2.2 Morphology and Fiber Diameter

To characterize the morphology of the electrospun fibers, samples were placed onto aluminum holders and sputter coated with gold for 60 s with a sputter current of 20 mA, before being studied under a Phenom Pro benchtop scanning electron microscope (SEM; Thermo Fisher, UK). Fiber diameter was measured from SEM images with ImageJ.

### 2.3 Physicochemical Characterization

Attenuated total reflectance (ATR)-Fourier-transform infrared spectroscopy (FTIR) spectra of electrospun samples were obtained by using a Spectrum 100 spectrometer (Perkin Elmer, UK). The spectra were collected with a resolution of 1 cm^–1^ in the wavenumber range of 4000–650 cm^–1^ at an average of 8 scans for each sample. Samples of approximately 0.2 cm × 0.2 cm were placed directly onto the ATR crystal, and pressure was exerted to guarantee contact.

X-ray diffraction (XRD) patterns of the samples and raw materials were obtained using a Miniflex 600 (Rigaku, Japan) diffractometer supplied with Cu Kα radiation (λ = 1.5418 Å). Samples of approximately 1 cm^2^ were placed in a glass sample holder. The patterns were recorded in the 2θ range 3° to 50° at a speed of 0.5° min^–1^. The generator voltage was set at 40 kV and the current at 15 mA.

Differential scanning calorimetry (DSC) analysis was performed using Q2000 DSC (TA Instruments, UK) at a temperature ramp of 10 °C min^–1^ from 25 to 225 °C. A sample of approximatively 5–7 mg was placed inside a nonhermetically sealed aluminum pan (T130425, TA Instruments, UK), with an empty aluminum pan as a reference. Oxygen-free nitrogen gas at a purge rate of 50 mL min^–1^ was supplied to the furnace throughout the process.

Thermogravimetric analysis was conducted using a TA Instruments Discovery instrument at a temperature ramp of 10 °C min^–1^ from 40 to 500 °C under a nitrogen purge of 25 mL min^–1^.

### 2.4 Surface Hydrophobicity

The contact angle (CA) of the samples was measured using the static sessile drop method with a contact angle goniometer (OCA40, DataPhysics, UK) equipped with a high-speed camera and a Cole Parmer micrometer syringe tip. A 2 μL water droplet was dispensed onto the electrospun patches, and its behavior on the fiber surface was recorded for 60 s using the high-speed camera. Images captured at specific time intervals were used for the analysis.

### 2.5 Encapsulation Efficiency and In Vitro Drug Release Kinetics

To quantify drug loading and encapsulation efficiency of electrospun patches, 1 mg of each fiber sample was dissolved in 4 mL of dichloromethane. The quantification of riluzole in resultant solutions was performed by a UV–visible spectrometer (Jenway, UK) at 263 nm, and the riluzole encapsulation efficiency within electrospun fibers was calculated using eq 1

1 $$EE\;\left(\%\right)=\frac{detected\;mass\;of\;riluzole\;in\;fiber}{initial\;mass\;of\;riluzole\;in\;feedstock}\times 100\%$$

To assess in vitro drug release kinetics, 10 mg fiber samples were placed in 5 mL of phosphate-buffered saline (PBS) (Sigma-Aldrich, UK) solution supplemented with 0.05% w/v sodium azide (NaN_3_) (Sigma-Aldrich, UK) as a preservative. The samples were placed in a shaking incubator (Incu-Shake, SciQuip, UK) at 37 °C and 120 rpm, and every 2 days, a 500 μL sample was taken, which was then replaced with the same volume of fresh PBS–NaN_3_ solution. The quantification of riluzole in resultant solutions was performed by a SpectraMax M2e microplate reader (Molecular Devices, UK) at 263 nm.

### 2.6 In Vitro Cell Experiments

#### 2.6.1 SH-SY5Y Cell Culture

SH-SY5Y cells (ATCC) were cultured in complete media (1:1 EMEM (Sigma-Aldrich, UK) and HAMS-F12 (Sigma-Aldrich, UK) media supplemented with 15% v/v fetal bovine serum (Sigma-Aldrich, UK), 1% v/v penicillin and streptomycin (Thermo Fisher, UK), 1% nonessential amino acids (Thermo Fisher, UK) and 2 mM l-glutamine) at 37 °C and 5% CO_2_. The cells were subcultured once a week and used within passages 5–12.

#### 2.6.2 Preparation of Riluzole Stock Solutions and Glutamate-Containing Media

A stock solution of riluzole (98%, Thermo Fisher, UK) was prepared by dissolving the powder in sterile dimethyl sulfoxide (DMSO). To prepare glutamate-containing media, l-glutamic acid monosodium salt monohydrate (glutamate; Sigma-Aldrich, UK) was dissolved to a final concentration of 100 mM in complete media.

#### 2.6.3 Optimization of the SH-SY5Y Glutamate-Induced Excitotoxicity Model

200 μL of SH-SY5Y cells at 5 × 10^4^ cells/mL was seeded in a flat-bottomed 96-well plate and incubated for 24 h, after which the complete medium was removed from each well. For optimization of glutamate-induced cytotoxicity, cells were incubated in media (200 μL) containing various concentrations of glutamate (0.1–100 mM) for 3, 24, or 72 h. To test the cytotoxicity of riluzole on glutamate-untreated SH-SY5Y cells, immediately before the experiment, riluzole stock solutions in DMSO were diluted in prewarmed complete media (1:1000). The cells were then incubated with riluzole-containing media (200 μL) for 3, 24, and 72 h. At the end of the incubation period, cell viability was quantified using the PrestoBlue Cell Viability reagent (Thermo Fisher, UK) according to the manufacturer’s instructions.

#### 2.6.4 Riluzole Treatment in the SH-SY5Y Glutamate-Induced Excitotoxicity Model

To test the effect of riluzole on glutamate-induced excitotoxicity, 200 μL of SH-SY5Y cells at 5 × 10^4^ cells/mL was seeded in a flat-bottomed 96-well plate and incubated for 24 h, after which the complete medium was removed from each well.

For co-administration assays, cells were treated simultaneously with riluzole and glutamate for 24 h. Immediately before the experiment, riluzole stock solutions in DMSO were diluted in a glutamate-containing medium (1:1000). The resultant riluzole- and glutamate-containing medium (200 μL) was added to each well, and the plate was incubated for a further 24 h.

For postadministration assays, cells were first exposed to the glutamate-containing medium for 24 h, following which the medium was removed. The cells were then incubated for an additional 24 h with a riluzole stock solution diluted in complete media (1:1000).

In both assays, “no treatment” control wells received complete media alone, while “vehicle”-treated cells received DMSO diluted in complete media (1:1000). “Cell death” control wells were treated with 70% ethanol five minutes before the end of the incubation period. At the end of the incubation period, cell viability was quantified using the PrestoBlue Cell Viability reagent (Thermo Fisher, UK).

#### 2.6.5 Biocompatibility Testing of Fiber Patches

500 μL of SH-SY5Y cells at 5 × 10^4^ cells/mL was seeded in a flat-bottomed 24-well plate and incubated for 24 h. Fiber patch samples (∼1 mg) containing 0%–5% w/v riluzole were cut into 6 mm diameter discs using a hole punch, sterilized for 20 min by UV radiation on each side, and introduced to the wells containing adhered cells with sterile tweezers. The plate was incubated for 3 days to measure cell viability. At the end of the incubation period, the fibers were removed, and cell viability was quantified using the PrestoBlue Cell Viability reagent (Thermo Fisher, UK).

#### 2.6.6 Cell Treatment with Fiber Patches in Glutamate-Induced Excitotoxicity

To verify the pharmacological effect of riluzole-loaded fiber patches, 500 μL of SH-SY5Y cells at 5 × 10^4^ cells/mL was seeded in a flat-bottomed 24-well plate and incubated for 24 h, after which the complete medium was removed and replaced with 500 μL of glutamate-containing media. Fiber patch samples (∼1 mg; 6 mm diameter discs) containing 0%, 0.25%, 0.5%, and 1% riluzole w/v were sterilized for 20 min by UV radiation on each side and introduced to the wells containing adhered cells with sterile tweezers. “Cells only” control wells received no fiber samples or glutamate and “cell death” control wells were treated with 70% ethanol 5 min before the end of the incubation period. Following 24 h incubation, the fibers were removed, and cell viability was quantified using the PrestoBlue Cell Viability reagent (Thermo Fisher, UK).

### 2.7 Immunostaining and Imaging

For immunohistological analysis, SH-SY5Y cells were seeded on sterile coverslips before being subjected to one of the treatments outlined in Sections 2.6.3 and 2.6.6. Following treatment, the cells on coverslips were washed twice with Dulbecco’s PBS (DPBS) for 10 min each to remove any remaining culture media. The cells were then fixed with 4% paraformaldehyde in DPBS (Fisher Scientific) for 20 min at room temperature (RT).

After they were fixed, the cells were washed three times with PBS for 10 min each. Next, the cells on coverslips were permeabilized using 0.1% (v/v) Triton X-100 in DPBS (DPBS-Tx) for 10 min at RT and subsequently washed twice for 10 min each. The coverslips were then incubated overnight at 4 °C in the dark with a solution containing rabbit anti-β-III tubulin primary antibodies and Alexa Fluor 488 Phalloidin (both at 1:1000, Invitrogen) in DPBS-Tx. The following day, coverslips were washed three times with DPBS for 10 min each and then incubated with Alexa Fluor 555 goat antirabbit secondary antibodies (1:1000, Invitrogen) overnight at 4 °C in the dark. On the final day, the immunostained coverslips were washed three times with DPBS for 10 min each and incubated with 4′,6-diamidino-2-phenylindole (DAPI) (1:500 in DPBS) for 15 min at RT in the dark. After a final brief DPBS wash, the stained coverslips were mounted onto slides using Fluoromount mounting media (Invitrogen, UK) and stored at 4 °C until imaged.

For imaging, a Nikon 90i fluorescence microscope with NIS Elements BR software using constant exposure, gain, and magnification (20–40×) was used to analyze the cell-seeded coverslips. Acquired images were exported to the NIH ImageJ-FIJI open-source imaging software, where they were processed for brightness and contrast.

### 2.8 Experimental Design and Statistics

For the statistical analysis of in vitro cell experiments, one-way ANOVA or two-way ANOVA, followed by Dunnett’s multiple comparison test was performed. All experiments were conducted with triplicate samples and with three technical replicates for cell lines (*n* = 3). Data are presented as mean ± standard deviation (SD). For each experiment, robust regression and outlier removal (ROUT) analysis was performed to identify and remove any outlier values, with *Q* = 1%. Differences are characterized as statistically significant if *P* < 0.05. The GraphPad Prism software (version 10.2.2) was used to perform all statistical calculations, as well as to plot all data.

## 3 Results and Discussion

### 3.1 Optimization of an In Vitro Glutamate-induced Excitotoxicity Model in SH-SY5Y Cells

There is limited research in the literature on developing an in vitro model for glutamate-induced excitotoxicity in SCI, and there appears to be no consensus on the optimal glutamate dose needed to replicate these conditions.^26,27^ In this study, high concentrations of glutamate (100 mM) were used to induce neuronal injury in SH-SY5Y cells, given their potential insensitivity to lower levels.^28^ In vivo, glutamate uptake and circulatory clearance result in lower extracellular concentrations. Additionally, past studies often measured glutamate levels in fluids rather than in tissues, resulting in inconsistent threshold values.^29^ Using higher concentrations in vitro reflects elevated glutamate conditions post-SCI, providing a more accurate model for studying neuroprotection and injury mechanisms.^29^ To optimize the glutamate-induced excitotoxicity model in SH-SY5Y cells, we tested a range of glutamate concentrations over three treatment durations (3, 24, and 72 h) (Figure S1a) and observed that 100 mM glutamate sufficiently lowered SH-SY5Y cell viability after 24 h exposure (Figures 1a–c and S1a) while preserving sufficient viable cells to support neural recovery processes.

**Figure 1:**
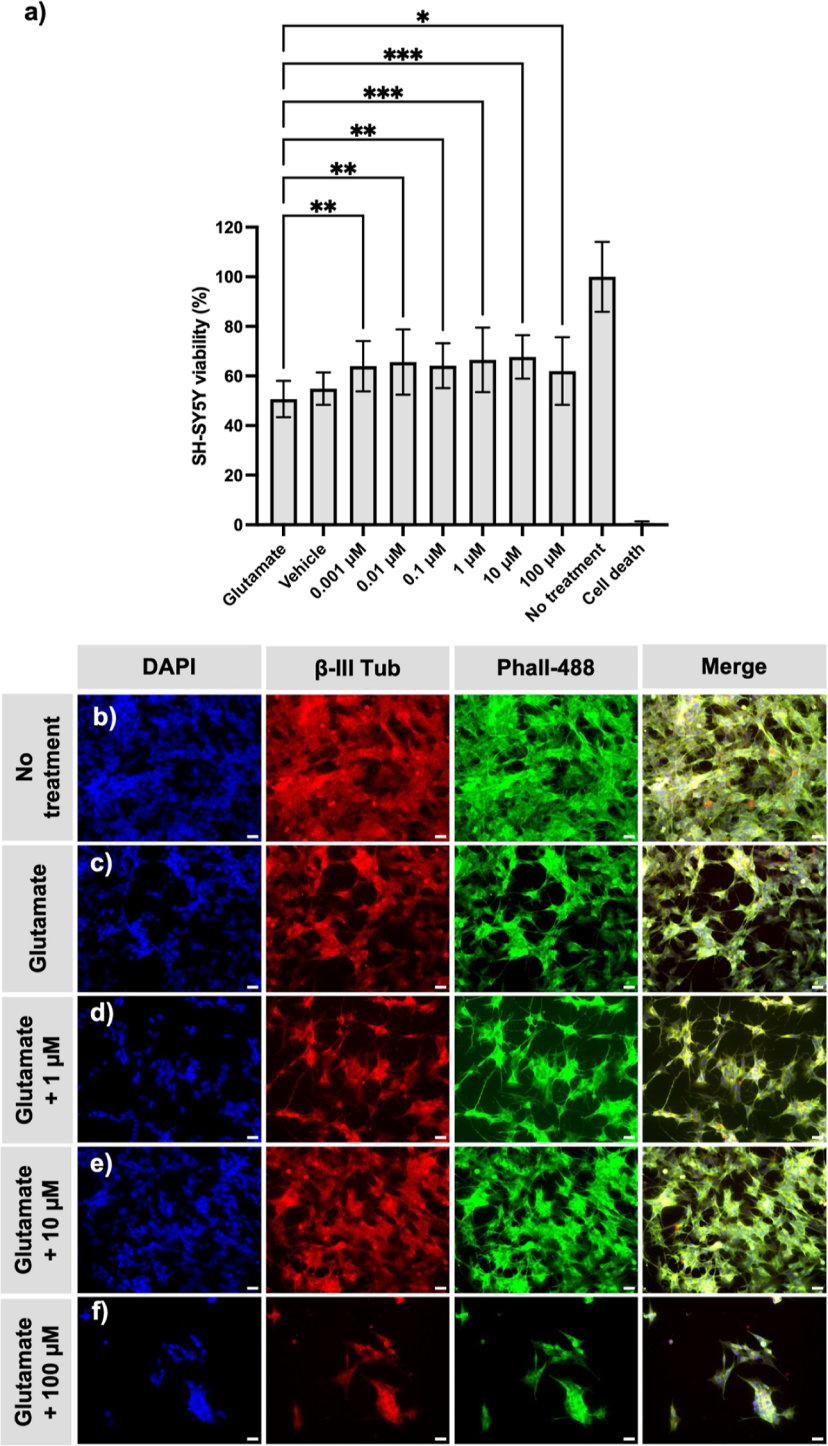
The effect of riluzole treatment on SH-SY5Y cell viability in a glutamate-induced cytotoxicity model. SH-SY5Y viability following co-administration of riluzole and glutamate over 24 h (a), where each riluzole concentration causes a significant increase in cell viability. Fluorescent microscopy images of DAPI and phalloidin-488 (Phall-488) stained and β-III tubulin (β-III tub) immunostained SH-SY5Y cells exposed to no treatment (b), 100 mM glutamate alone (c), and glutamate-exposed neurons treated with 1 μM (d), 10 μM (e), and 100 μM (f) riluzole. Note the drop in cell density in glutamate exposed cultures (c) and the increased density, especially in 10 μM riluzole-treated cells (e). SH-SY5Y cells exposed to 100 μM riluzole had fewer cell processes and tended to clump as clusters (f, merge at right). “Glutamate”: control (cells treated with 100 mM glutamate in media), “vehicle”: cells treated with DMSO, in which riluzole was dissolved, “no treatment”: positive control (cells incubated without glutamate or riluzole), and “cell death”: negative control (cells treated with 70% ethanol for 5 min before PrestoBlue administration). **P ≤ 0.01, ****P ≤ 0.0001. Scale bar = 25 μm.

Glutamate acts as a fast-acting neurotransmitter, rapidly inducing cell depolarization and leading to ion imbalances that culminate in cell death.^30^ The results suggest that co-administration of riluzole is effective in mitigating glutamate-induced cytotoxicity, as all tested concentrations of riluzole exhibited significantly higher cell viability (Figure 1a). Specifically, riluzole concentrations of 1 and 10 μM appear to be optimal, while higher doses induced drug-related cytotoxicity (Figures 1d–f and S1b). These experiments indicate that administration of riluzole is capable of rescuing cells subjected to glutamate-induced injury, such as that experienced during the active secondary injury stage, possibly by readjusting the disrupted sodium ion balance.^31^

However, this mechanism is effective only when riluzole is administered simultaneously with glutamate, as postadministration fails to reverse the damage already inflicted (Figure S2). In a clinical context, the co-administration approach is plausible, as patients with spinal cord injury typically undergo immediate surgical intervention,^32^ which provides an opportunity to insert a riluzole-loaded implant during the active secondary spinal cord injury phase. Other studies have demonstrated the benefits of riluzole in decreasing the glutamate excitotoxicity. For instance, Chang et al. tested riluzole in an organotypic rat spinal cord injury model, co-administering it with sulforaphane. Their findings showed that a 5 μM riluzole dose, which is comparable to the dosage in our study, resulted in a decrease in glutamate levels.^33^ Similarly, in a study conducted by Nicholson et al., riluzole was administered to rats following nerve root injury, and the result showed that the treated animals exhibited less axonal swelling and decreased neuronal excitability 7 days after a single dose.^34^ Additionally, Hama and Sagen found that administering riluzole in rats following SCI had an antinociceptive effect, suggesting its potential to alleviate neuropathic SCI pain by inhibiting excitatory pathways, although the exact mechanism remains unclear.^35^

### 3.2 Electrospun Implant Optimization

#### 3.2.1 Manufacturability and Morphology

The proposed riluzole-loaded patch facilitating the local delivery of the drug to the spinal cord could be incorporated as a lining to a commercially available dural substitute (such as DuraGen) or as a standalone biodegradable implant. In this study, we opted for a single nozzle electrospinning of a simple polymer–drug blend formulation to simplify the fabrication, easing potential experimental scale-up for future preclinical studies. The implant production process exhibited remarkable stability with no manual adjustments needed throughout the electrospinning process, which is advantageous for scalability in eventual commercial applications. Additionally, varying drug concentration within the patches required no further process optimization, suggesting that the therapeutic dose could be easily adapted to specific application needs.

PCL was selected due to its excellent surgical handling properties^25^ and relatively long biodegradation profile, providing a scaffold for migrating dural cells to colonize and integrate into the dural layer.^24^ In the context of spinal injury, PCL has been previously used to deliver therapeutics such as uric acid^36^ or glial cell-derived neurotrophic factor.^37^ Target riluzole loading within PCL patches was identified through a preliminary 72 h dose–response study in SH-SY5Y cells. Following a three day incubation, fiber patches with drug loading concentrations above 1% w/v were found to be significantly cytotoxic (Figure 2). Consequently, for all subsequent experiments, only fiber patches with 0%, 0.25%, 0.5%, and 1% w/v riluzole loadings were used.

**Figure 2:**
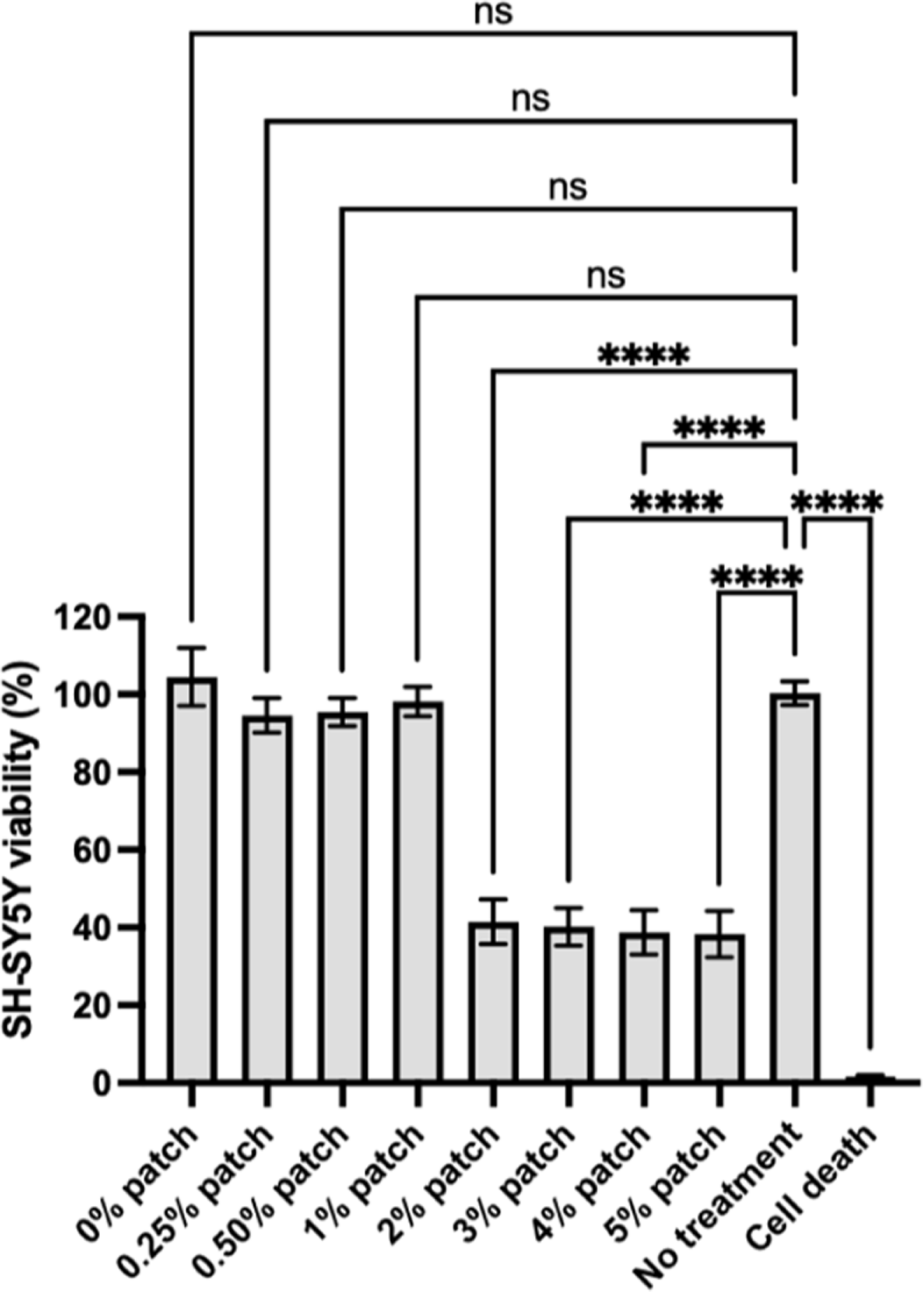
SH-SY5Y cell viability after a 3 day incubation with fibers at different riluzole loadings. Fibers containing 2%, 3%, 4%, and 5% w/v riluzole significantly decrease SH-SY5Y cell viability. “No treatment”: positive control (cells incubated without any fiber samples) and “cell death”: negative control (cells treated with 70% ethanol for 5 min) before PrestoBlue administration. ****P ≤ 0.0001, ns (nonsignificant).

The produced patches were easy to handle with tweezers and cuttable using surgical scissors (Figure 3a), demonstrating their suitability for intraoperative use. Dural implants should be flexible and easy to manipulate, and the ability to cut the patches into different shapes and sizes to match the injury site is crucial.^38^

**Figure 3:**
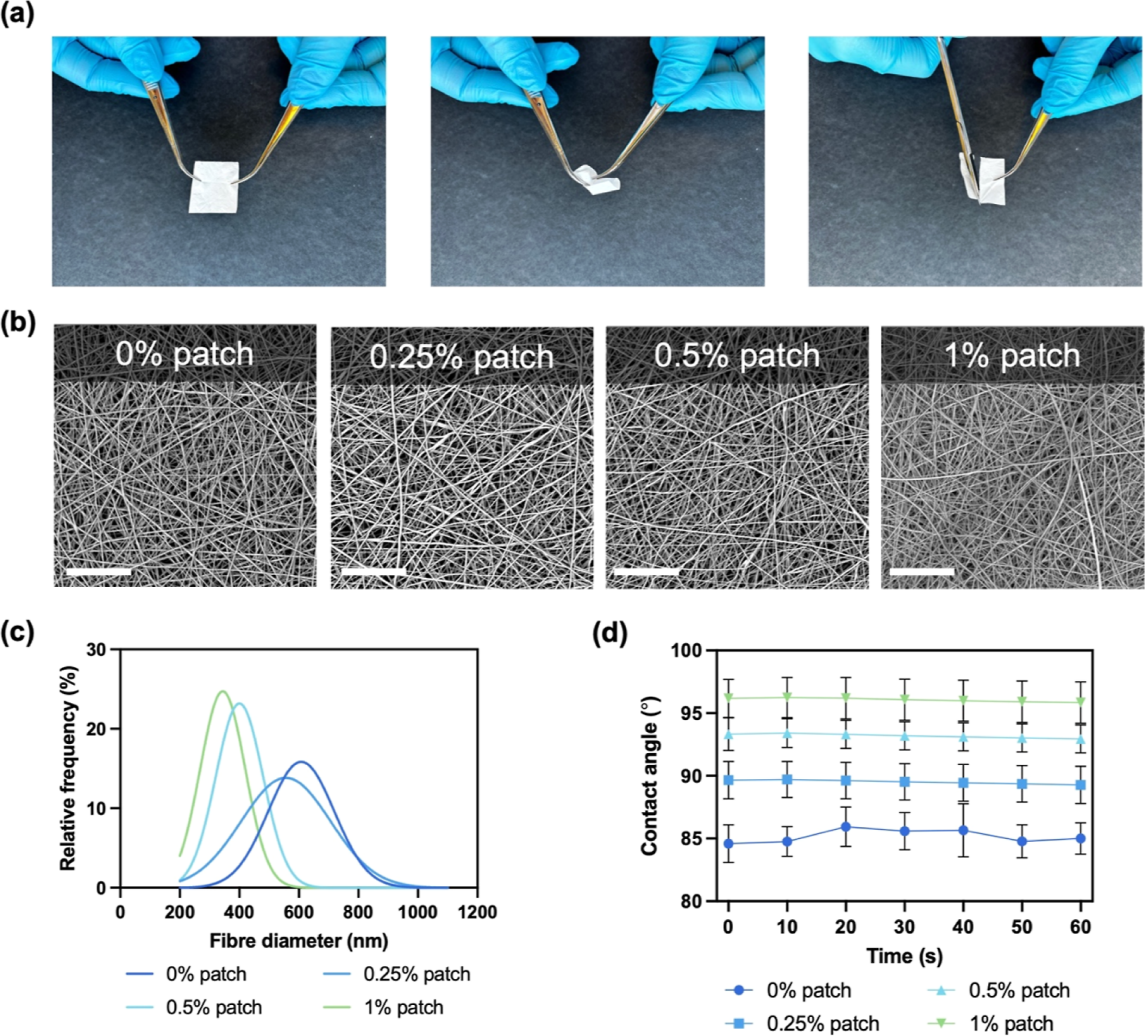
Summary of fiber patch properties. (a) Photographs of fiber patch handling mimicking surgical conditions, (b) scanning electron micrographs of 0%, 0.25%, 0.5%, and 1% w/v (left to right) riluzole-loaded fiber patches, (c) unimodal distribution of fiber diameter measurements, and (d) contact angle measurements. Scale bar = 30 μm.

SEM images of the patches (Figure 3b) show the successful production of fibers with no obvious defects, demonstrating the stability of the electrospinning process. All patches exhibited a unimodal distribution of the fiber diameter. Blank PCL fiber patches showed the highest fiber diameter with 608 ± 111 nm, and with increasing concentrations of riluzole, the fiber diameter decreased, with 556 ± 150 nm, 400 ± 79 nm, and 344 ± 76 nm for 0.25%, 0.5%, and 1% w/v riluzole, respectively (Figure 3c). This may be attributed to riluzole acting as a plasticizer, breaking up chain entanglement and resulting in a thinner polymer jet before the deposition of the resulting fibers.^39^ Alternatively, riluzole may increase the number of charge carriers in the polymer solution, causing the whipping stage to occur earlier. This can result in a longer stretching period and therefore a smaller fiber diameter.^39^ A study conducted by Johnson et al. observed a similar trend when producing PLLA fiber patches encapsulating riluzole, where the presence of riluzole led to a decrease in fiber diameter.^39^ Adjusting the polymer concentration could contribute to restoring the fiber diameter after the incorporation of riluzole.^40^

To assess the hydrophobicity of the patches, the contact angle of a water droplet placed on the surface of the electrospun samples was measured over 60 s. Blank PCL patches showed hydrophobicity, with a contact angle of 85 ± 1° remaining constant over time (Figure 3d). The contact angle for drug-loaded fiber patches modestly increased with higher drug concentrations, likely due to the lipophilicity of riluzole.^41^ Hydrophobicity of the samples is desired since the patch should help prevent cerebrospinal fluid leakage.

#### 3.2.2 Physicochemical Characterization

Electrospinning promotes the formation of amorphous solid dispersions by enabling rapid solvent evaporation, which inhibits drug crystallization and stabilizes the amorphous phase, thus enhancing solubility and bioavailability.^42^ The FTIR spectrum of raw riluzole shows two peaks at around 3363 cm^–1^ and 3275 cm^–1^, attributed to N–H stretching vibration (Figure 4a) and confirming the presence of the primary amine group in the structure of riluzole.^43^ The peaks at 815 cm^–1^ and 870 cm^–1^ correspond to the C–H bending vibration in the aromatic ring. The peaks at 1462 cm^–1^ resulting from C=C stretching vibration, at 1642 cm^–1^ resulting from C=N stretching vibration, and at 1541 cm^–1^ resulting from C–H in plane bending vibration can all be ascribed to the vibrations from the benzothiazole aromatic ring in the riluzole structure.^43^ The spectrum of the blank PCL fiber patch shows a sharp characteristic peak at around 1724 cm^–1^ arising from the C=O stretching vibration in the carboxylic acid group.^44^ Two bands observed at 2939 cm^–1^ and 2868 cm^–1^ can be attributed to the C–H stretching vibration in the structure of PCL. The peak at around 1164 cm^–1^ results from the C–O stretching vibration.^44^ The spectra of riluzole-loaded electrospun fiber patches share a high similarity with the blank PCL fiber patch, and the characteristic absorption peaks of riluzole are not visible, showing that riluzole was successfully encapsulated within the fibers.

**Figure 4:**
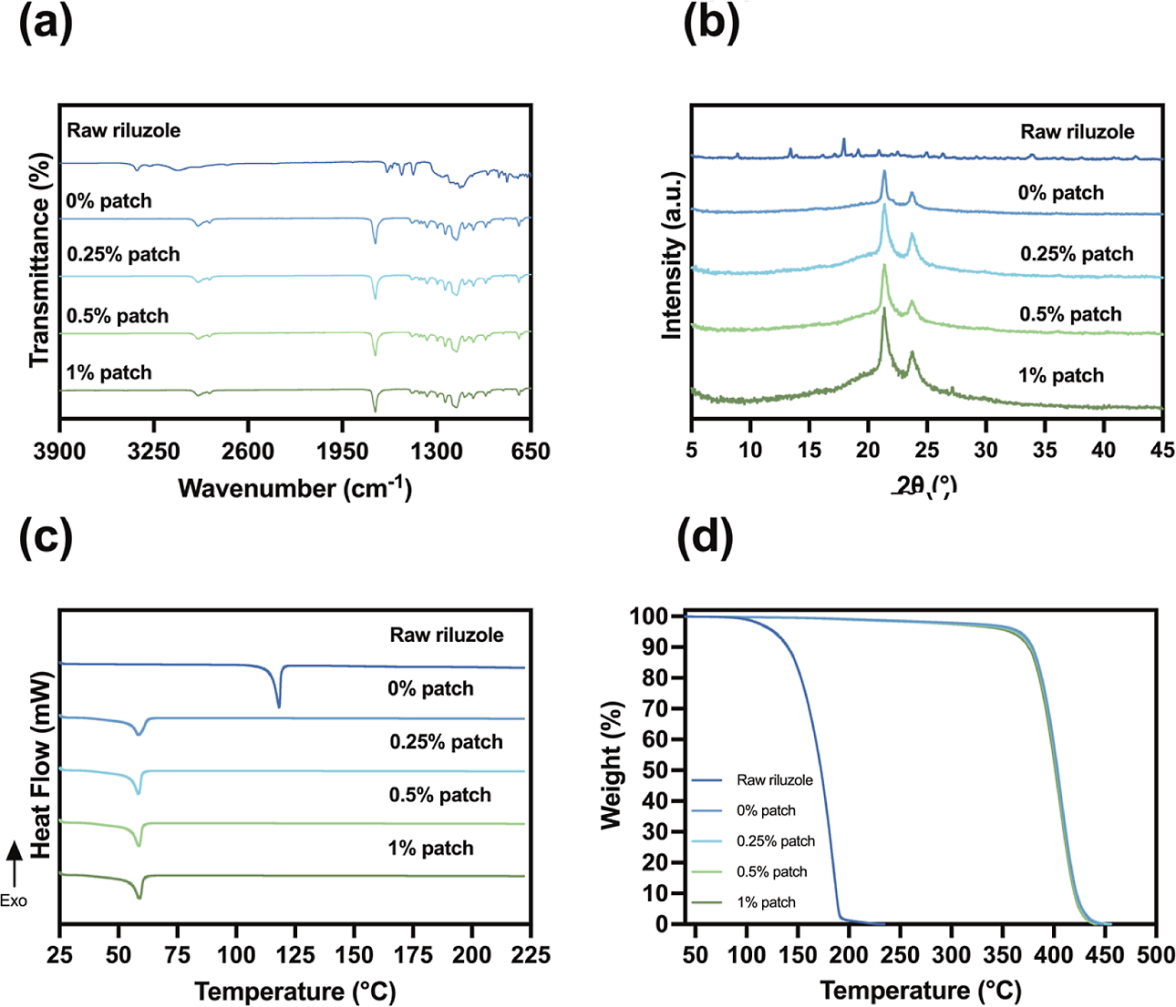
Physicochemical characterization of riluzole and PCL patches containing varying concentrations of the drug. FTIR (a), XRD (b), and DSC (c) data confirm the mostly amorphous nature of patches. TGA (d) shows no residual solvent was present postelectrospinning.

In X-ray diffractograms (Figure 4b), riluzole displays characteristic sharp Bragg reflections at 2θ = 8.9°, 13.4°, 18.0°, 19.2°, 21.0°, 22.5°, 25.0°, 26.4°, 34.1°, and 43.5°, suggesting the highly crystalline nature of riluzole, which is consistent with the literature.^45,46^ Blank PCL fiber patches exhibited only two sharp peaks at around 22.0° and 24.0° (Figure 4b) and some halos can be observed, revealing the semicrystalline nature of PCL.^47^ XRD patterns of riluzole-loaded fiber patches are highly similar to those of the blank sample, with no characteristic peaks of raw riluzole observed. This confirms the successful incorporation of riluzole into the patches, with the drug dispersed throughout the fibrous matrix in an amorphous state, which can theoretically enhance solubility by improving bioavailability and drug release.^48,49^ The absence of characteristic peaks of riluzole may also be attributed to the relatively low drug content, which is less than 2% w/v.

DSC thermograms (Figure 4c) show an endothermic peak indicating the melting point in both raw materials and electrospun fiber patches. Riluzole exhibits a single sharp peak at around 117 °C, corresponding to its reported melting point of 119 °C.^46^ Blank PCL fibers exhibit a slightly broad peak at around 57 °C, similar to that of raw PCL, which is observed at approximately 59 °C, as has been reported previously.^50^ The riluzole-loaded fiber patches show no difference compared to those of blank PCL fiber patches, and the sharp peak belonging to raw riluzole is not visible, further demonstrating the successful incorporation of riluzole into the fibers. Moreover, the variations among fibers with different concentrations of riluzole are not significant. Each sample exhibits a single peak, indicating that the inherent structure of raw PCL remains intact and that the polymer–drug complexes function as a single system rather than as separate components.^51^

The TGA curve (Figure 4d) of riluzole shows a sharp decline between 100 and 200 °C, suggesting that riluzole begins to degrade at around 103 °C and is almost completely degraded by 196 °C, with a total weight loss of up to 95%. For blank PCL fiber patches, degradation initiates at around 304 °C and progresses until approximately 440 °C with a similar weight loss of 95%, consistent with the findings of Ravichandran et al.^52^ The degradation behavior of riluzole is not evident in the TGA curves of drug-loaded fiber patches, which may suggest effective encapsulation of riluzole within the electrospun fibers and the formation of stable drug–polymer complexes. Furthermore, the absence of degradation behavior may also be attributed to the relatively low drug content, as noted in the X-ray diffractograms (Figure 4b). The TGA curves of the resulting electrospun fiber patches with various riluzole concentrations highly coincide, indicating that the structure of PCL is not damaged during the electrospinning process and the incorporation of riluzole does not significantly affect the degradation behavior of PCL fiber patches. These observations are in agreement with the XRD and DSC data.

#### 3.2.3 Drug Release Kinetics and Cytocompatibility

Encapsulation efficiency measurements showed that fiber patches containing 0.25% w/v riluzole exhibited an encapsulation efficiency of 54.02 ± 5.59%. As the riluzole loading within the fibers increased, the encapsulation efficiency improved, reaching 67.27 ± 12.89% and 81.08 ± 8.51% for 0.5% and 1% w/v riluzole-loaded fiber patches, respectively. This trend can be attributed to a greater probability of drug loss when minimal drug amounts are dissolved in solution.

A drug release study was performed over 52 days (Figure 5). Because PCL hydrolyzes slowly (over 3 months),^53^ riluzole was expected to release from fibers through diffusion. All formulations exhibited a burst release of riluzole within 24 h. Patches containing 0.25%, 0.5%, and 1% w/v riluzole showed a burst release of 70.5 ± 3.5, 160.6 ± 0.9, and 177.0 ± 2.2 μg, respectively, followed by a slow sustained increase to 97.7 ± 2.6, 294.2 ± 2.6, and 417.6 ± 2.8 μg, respectively, after 52 days. At these time points, we observed a significant difference in the cumulative amount released across all tested formulations (Figure S2). This release profile aligns with findings presented in Figure 1, demonstrating that co-administration of riluzole with glutamate is most effective at mitigating excitotoxicity, and therefore, a burst release is desired. Over time, the sustained patch release of riluzole continued to protect against prolonged neuronal exposure to elevated extracellular glutamate levels. This corresponds with observations from other studies where riluzole administered immediately after injury was shown to be most effective. One study, conducted by Wu and colleagues, explored the effects of intraperitoneal riluzole injection in rats administered one or three hours after SCI. The results indicated that riluzole administered one hour postinjury significantly reduced inflammation and apoptosis. Pharmacokinetic data showed that riluzole reached the spinal cord within 15 min of the injection, explaining its rapid neuroprotective effects.^54^ In another study conducted by Wu et al., a single dose of riluzole administered immediately post-SCI decreased the expression levels of interleukin-1β mRNA within 6 h and decreased the activation of various immune cells within 1 day.^55^ Additionally, riluzole-treated rats demonstrated improved motor function after 6 weeks compared to control rats.^55^ As these findings highlight the importance of an immediate administration of riluzole for optimal recovery, the observed burst release of riluzole from the produced fibers is highly desirable for the mitigation of neuronal death in SCI damage.

**Figure 5:**
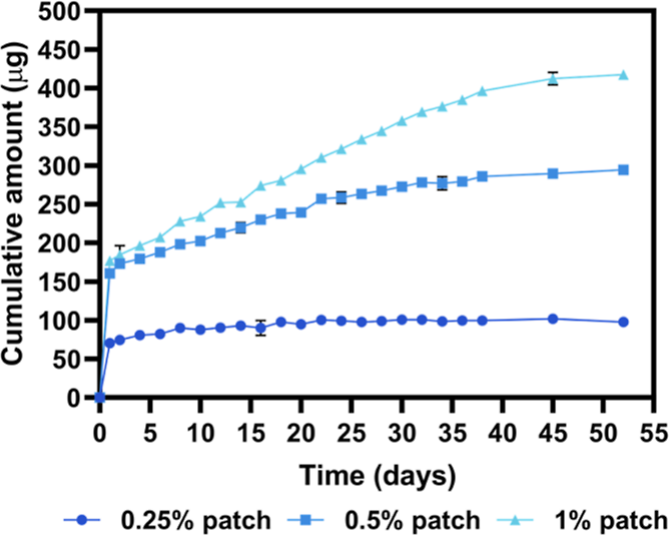
Drug release study. Cumulative amount of riluzole released (μg) over 52 days from 0.25%, 0.5%, and 1% w/v fiber patches. All release profiles show a burst release of riluzole within the first 24 h, followed by a slow sustained increase over the next 52 days. Data are presented as mean ± SD (*n* = 3).

#### 3.2.4 Pharmacological Effect in the Glutamate-Induced SH-SY5Y Excitotoxicity Model

To test whether riluzole remains pharmacologically active after being processed into the fibers, the viability of SH-SY5Y cells was measured following a 24 h incubation with 100 mM glutamate and fiber patches at various riluzole concentrations (Figure 6). Blank PCL fiber patches (0% patch) showed no significant difference in cell viability compared to the glutamate control group (46 ± 4%), where cells were exposed to glutamate without fiber patches. In contrast, fibers loaded with 0.25%, 0.5%, and 1% w/v riluzole significantly improved cell viability, with the highest viability increase seen in cells incubated with 1% riluzole patches (72 ± 1%), followed by the 0.5% patch (62 ± 3%) and the 0.25% patch (60 ± 5%).

**Figure 6:**
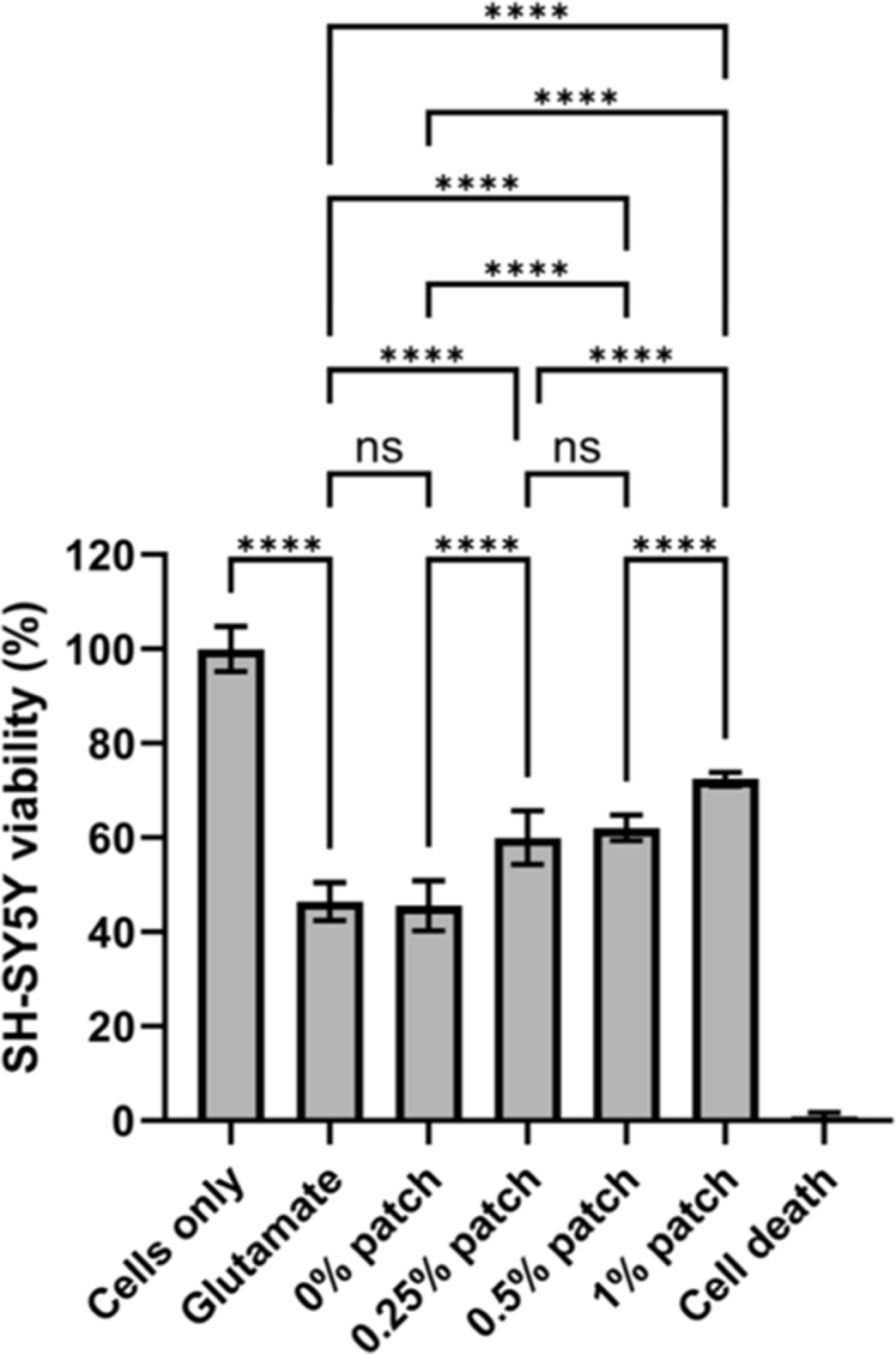
SH-SY5Y cell viability after a 24 h incubation with glutamate and riluzole-loaded fibers at different concentrations. All riluzole-loaded fibers show a significant increase in cell viability, whereas blank PCL fibers show no difference in cell viability compared to the control. “Glutamate”: control (cells treated with 100 mM glutamate in media), “cells only”: positive control (cells incubated without glutamate or fibers), and “cell death”: negative control (cells treated with 70% ethanol for 5 min before PrestoBlue administration). ****P ≤ 0.0001.

The results were further confirmed with fluorescence microscopy, where SH-SY5Y cells treated with a blank PCL fiber (0% patch) formed dense colonies, suggesting that the implant itself is biocompatible (Figure 7a). In contrast, cells insulted with glutamate showed a significant reduction in density (Figure 7b), while glutamate-insulted SH-SY5Y cells treated with 0.25% exhibited dense aggregates similar to those of the control sample (0% patch).

**Figure 7:**
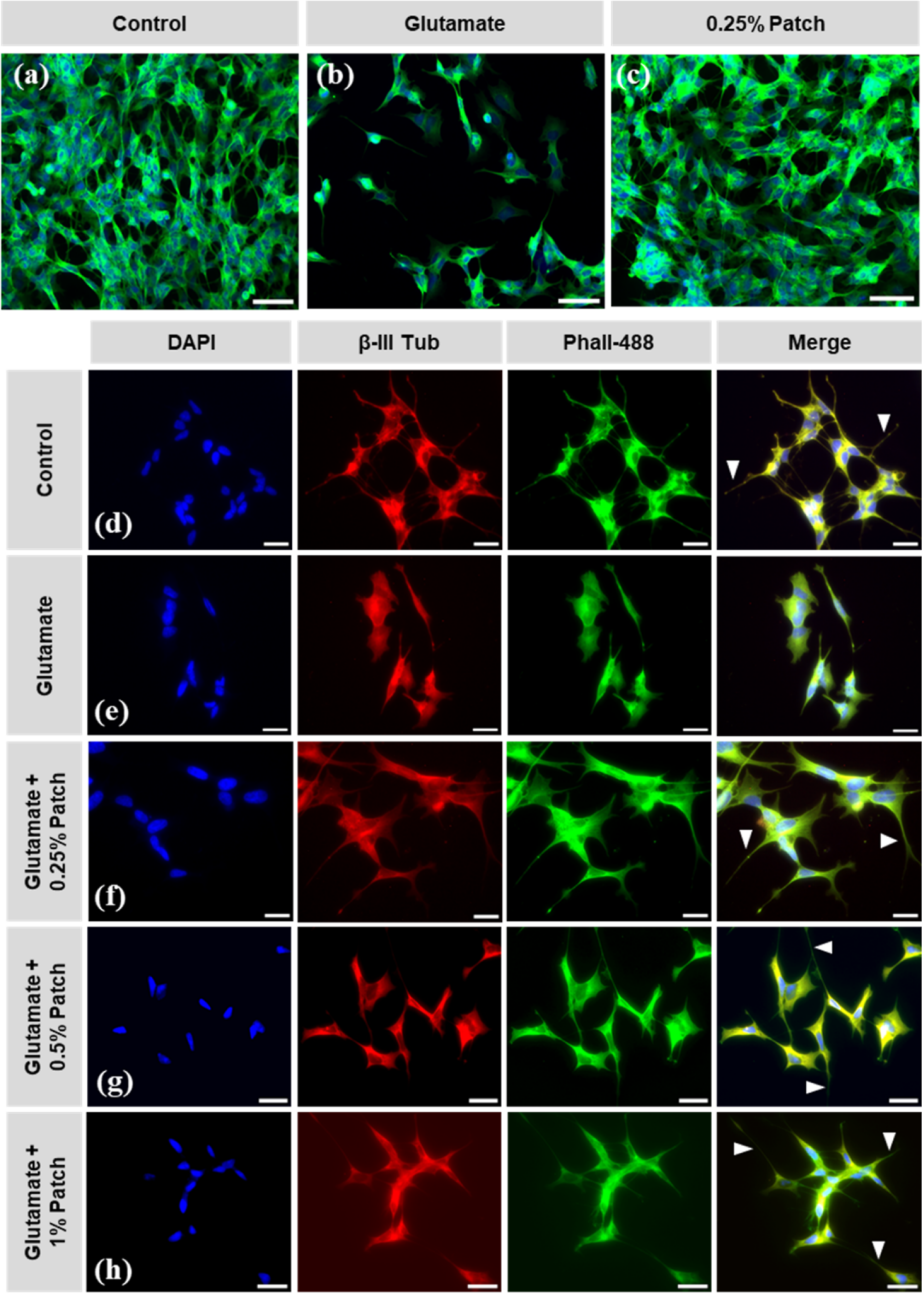
Fluorescence microscopy of SH-SY5Y cells after a 24 h incubation with glutamate and 0.25% w/v riluzole-loaded fibers. (a) SH-SY5Y cells grown in media from blank patches (control) formed dense colonies while cells exposed to (b) glutamate were less densely aggregated. Glutamate-exposed cells treated with riluzole (0.25% loaded patch) showed dense aggregates similar to the controls (c). Morphologically, SH-SY5Y cells treated with control (0%) patches possessed an elongate morphology extending 1–3 neurites (arrowheads) (d), whereas glutamate-exposed cells (e) were more cuboidal in shape. Cells treated with 0.25% (f), 0.5% (g), and 1% (h) riluzole-loaded patches exhibited a similar morphology to control cells (d), extending large numbers of long thin neurites (arrowheads in f–h). Scale bars in (a–c) = 50 μm and (d–h) = 25 μm.

Imaging of cell morphology showed that SH-SY5Y cells immunostained positively for β-III tubulin across all groups (Figure 7d–h) and cells exposed to media from 0% patches displayed a typical elongate neuron-like morphology with each cell typically extending 1–3 neurites (Figure 7d). In contrast, cells exposed to glutamate were less dense and demonstrated a more cuboidal-like morphology with fewer and shorter neurites (Figure 7e). Following treatment with 0.25% (Figure 7f), 0.5% (Figure 7g), and 1% (Figure 7h) patches, the cells possessed a mainly elongate morphology with long neurites similar to those of the control 0% patch group. This study confirmed that riluzole remains pharmacologically active postprocessing and still effectively counteracts glutamate-induced cytotoxicity, even at the lowest effective dose tested in the study (0.25% w/v).

## 4 Conclusion

In this study, we successfully produced electrospun fiber patches encapsulating riluzole, which was shown to remain pharmacologically active postprocessing, increasing SH-SY5Y cell viability in a model of glutamate-induced excitotoxicity. Physicochemical characterization of the fiber patches showed that riluzole was successfully encapsulated within PCL fibers in its amorphous form, improving its solubility. Encapsulated riluzole was released rapidly within the first 24 h, indicative of a burst release, which was then followed by a sustained release over a month. This biphasic release pattern is especially attractive for clinical translation, having the potential to counteract excessive glutamate-induced activation of injured neurons close to the injury site in the days after injury and in so doing reducing secondary injury-mediated cell death.

## Supplementary Material



## Notes

Optimization of the glutamate-induced SH-SY5Y cytotoxicity model; effects of post-treatment with riluzole on cell viability in that model; and cumulative riluzole release (μg) from 0.25%, 0.5%, and 1% w/v fiber patches over 1 and 52 days (PDF)
