# Supplementary material for: Neuroprotective Riluzole-Releasing Electrospun Implants for Spinal Cord Injury
Source: Mol Pharm. 2025 May 16;22(6):2905–16. doi: 10.1021/acs.molpharmaceut.4c01270 (PMC12135059; doi:10.1021/acs.molpharmaceut.4c01270)
Supplement: Supplementary file 1 [file mp4c01270_si_001.pdf]

Supplementary information

Neuroprotective riluzole-releasing electrospun implants for spinal cord injury

Mathilde M. Ullrich[1], Bhavana Pulipaka[2], Jing Yin[1], Jana Hlinková[1,2] Fangyuan Zhang[1], Michael Chan[1], Fergal J. O'Brien[3,4], Adrian Dervan[3,4]*, Karolina Dziemidowicz[1]*

[1] Department of Pharmaceutics, UCL School of Pharmacy, London, United Kingdom
[2] Institute of Experimental Medicine, Czech Academy of Sciences, Prague, Czech Republic
[3] Tissue Engineering Research Group, Department of Anatomy & Regenerative Medicine, Royal College of Surgeons in Ireland (RCSI), Dublin, Ireland
[4] Advanced Materials and Bioengineering Research Centre (AMBER), RCSI & TCD, Dublin, Ireland

*Corresponding authors: adriandervan@rcsi.ie, k.dziemidowicz@ucl.ac.uk

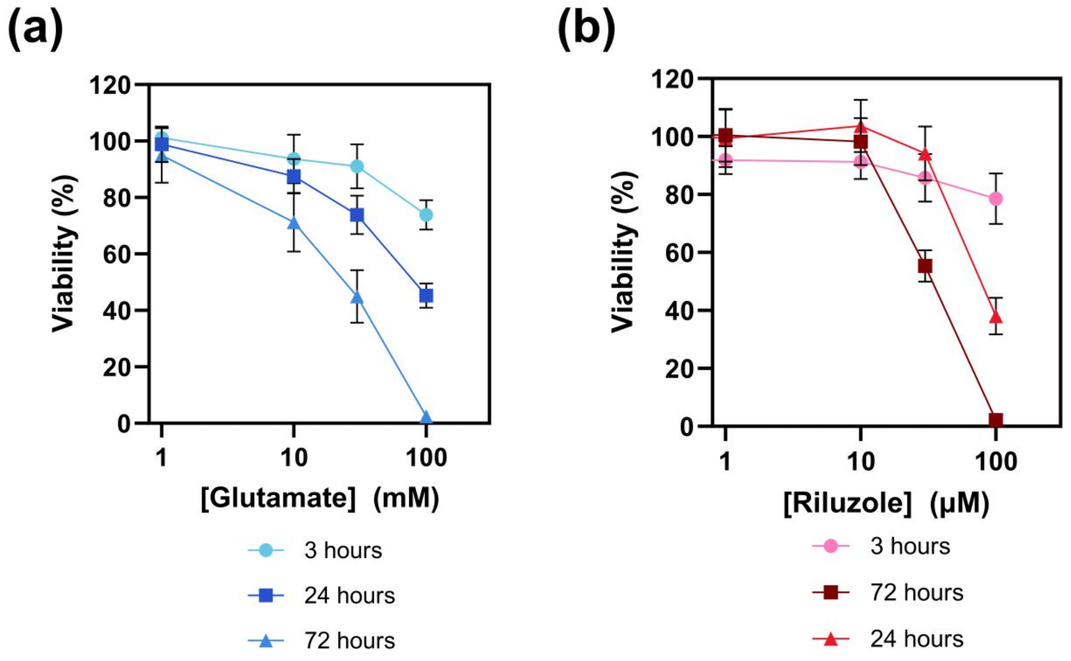

Figure S1. Optimisation of the glutamate-induced cytotoxicity model. The effect of concentration and treatment duration on SH-SY5Y cells treated with glutamate (a) and riluzole (b).

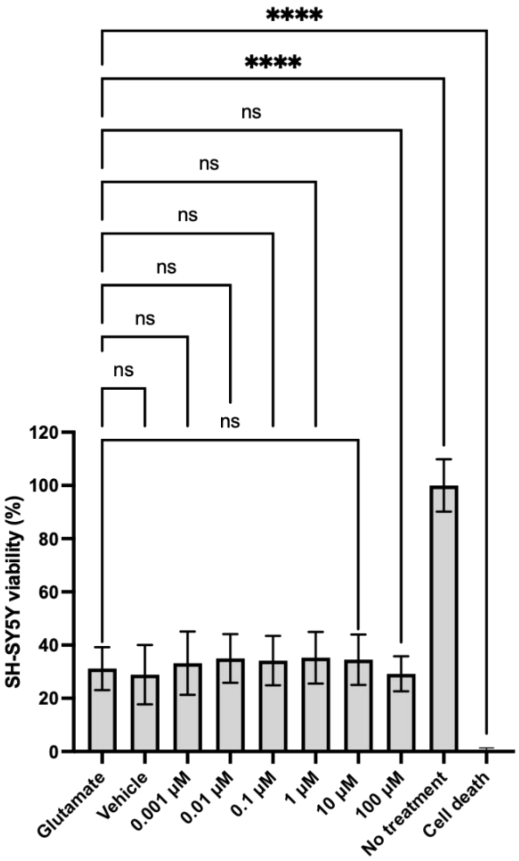

Figure S2. The effect of post-administration riluzole treatment on SH-SY5Y cell viability in a glutamate-induced cytotoxicity model. "Glutamate": control (cells treated with 100 mM glutamate in media), "Vehicle": cells treated with DMSO, in which riluzole was dissolved, "No treatment": positive control (cells incubated without glutamate or riluzole), "Cell death": negative control (cells treated with 70% ethanol for 5 minutes before PrestoBlue administration). $^{**}P \leq 0.01$, $^{****}P \leq 0.0001$, ns (non-significant).

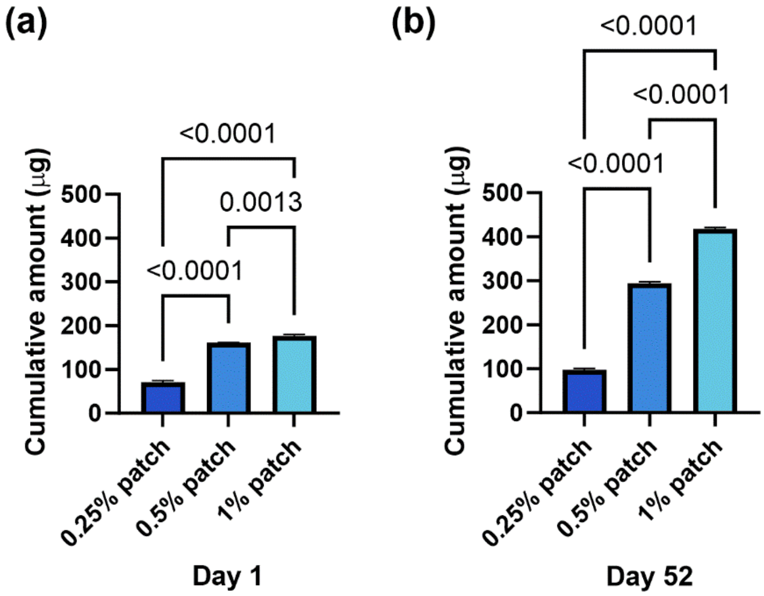

Figure S3. Comparison of cumulative amount of riluzole released (µg) over 1 (a) and 52 days (b) from 0.25%, 0.5% and 1% w/v fibre patches. Data are presented as mean ± SD (n=3).
